# Versatile SARS-CoV-2 Reverse-Genetics Systems for the Study of Antiviral Resistance and Replication

**DOI:** 10.3390/v14020172

**Published:** 2022-01-18

**Authors:** Ulrik Fahnøe, Long V. Pham, Carlota Fernandez-Antunez, Rui Costa, Lizandro René Rivera-Rangel, Andrea Galli, Shan Feng, Lotte S. Mikkelsen, Judith M. Gottwein, Troels K. H. Scheel, Santseharay Ramirez, Jens Bukh

**Affiliations:** 1 Copenhagen Hepatitis C Program (CO-HEP), Department of Infectious Diseases, Copenhagen University Hospital, 2650 Hvidovre, Denmark; ulrik@sund.ku.dk (U.F.); pham@sund.ku.dk (L.V.P.); carlota.fernandez.antunez@regionh.dk (C.F.-A.); rcosta@sund.ku.dk (R.C.); lizandro.rene.rivera.rangel@regionh.dk (L.R.R.-R.); andrea.galli@regionh.dk (A.G.); sfeng@sund.ku.dk (S.F.); lotte.scheibelein.mikkelsen@regionh.dk (L.S.M.); jgottwein@sund.ku.dk (J.M.G.); tscheel@sund.ku.dk (T.K.H.S.); santseharayra@sund.ku.dk (S.R.); 2 Department of Immunology and Microbiology, Faculty of Health and Medical Sciences, University of Copenhagen, 2200 Copenhagen, Denmark

**Keywords:** SARS-CoV-2, polymerase, remdesivir, replicon, RNA virus, molecular clone, GFP, nanoluciferase

## Abstract

The COVID-19 pandemic continues to threaten healthcare systems worldwide due to the limited access to vaccines, suboptimal treatment options, and the continuous emergence of new and more transmissible SARS-CoV-2 variants. Reverse-genetics studies of viral genes and mutations have proven highly valuable in advancing basic virus research, leading to the development of therapeutics. We developed a functional and highly versatile full-length SARS-CoV-2 infectious system by cloning the sequence of a COVID-19 associated virus isolate (DK-AHH1) into a bacterial artificial chromosome (BAC). Viruses recovered after RNA-transfection of in vitro transcripts into Vero E6 cells showed growth kinetics and remdesivir susceptibility similar to the DK-AHH1 virus isolate. Insertion of reporter genes, green fluorescent protein, and nanoluciferase into the ORF7 genomic region led to high levels of reporter activity, which facilitated high throughput treatment experiments. We found that putative coronavirus remdesivir resistance-associated substitutions F480L and V570L—and naturally found polymorphisms A97V, P323L, and N491S, all in nsp12—did not decrease SARS-CoV-2 susceptibility to remdesivir. A nanoluciferase reporter clone with deletion of spike (S), envelope (E), and membrane (M) proteins exhibited high levels of transient replication, was inhibited by remdesivir, and therefore could function as an efficient non-infectious subgenomic replicon system. The developed SARS-CoV-2 reverse-genetics systems, including recombinants to modify infectious viruses and non-infectious subgenomic replicons with autonomous genomic RNA replication, will permit high-throughput cell culture studies—providing fundamental understanding of basic biology of this coronavirus. We have proven the utility of the systems in rapidly introducing mutations in nsp12 and studying their effect on the efficacy of remdesivir, which is used worldwide for the treatment of COVID-19. Our system provides a platform to effectively test the antiviral activity of drugs and the phenotype of SARS-CoV-2 mutants.

## 1. Introduction

The coronavirus disease 19 (COVID-19) pandemic caused by the severe acute respiratory syndrome coronavirus 2 (SARS-CoV-2) has burdened national healthcare systems causing millions of deaths [1]. SARS-CoV-2 belongs to the *Coronaviridae* family and *Betacoronavirus* genus, which includes other human coronaviruses, such as the viruses causing severe acute respiratory syndrome (SARS) and Middle East respiratory syndrome (MERS) [2]. SARS-CoV and MERS-CoV caused outbreaks of severe respiratory diseases previously [3]. The first known cases of SARS-CoV-2 were reported in Wuhan, China and subsequently the number of infected individuals rapidly increased worldwide [4]. The outcome of SARS-CoV-2 infection varies from mild symptoms to severe respiratory distress and other complications that can lead to death [4]. 

SARS-CoV-2 contains a positive-sense single-stranded RNA genome of around 30 kb that consists of several open reading frames (ORFs). The first ORF (ORF1ab) encodes 16 nonstructural proteins (nsp) that are required for viral replication. Structural and accessory proteins are encoded by ORFs forming subgenomic mRNAs and viral RNA synthesis is regulated by transcription regulatory sequences (TRS), which are located upstream of most ORFs [5]. The viral structural proteins are the spike (S), envelope (E), nucleocapsid (N), and membrane (M) proteins.

Infectious full-length genomic SARS-CoV-2 clones are useful tools to investigate the fundamental processes of the viral life cycle that can lead to the identification of novel treatment targets, including the study of the role of specific mutations in antiviral resistance or immune evasion. On the other hand, non-infectious subgenomic systems (or replicons) are useful tools for the study of viral replication and drug efficacy with reduced biosafety risks [6,7]. 

The large coronavirus genome hampers the development of such reverse-genetics systems. Moreover, the viral genome contains toxic elements for bacteria, which are the most used cloning hosts, thus complicating plasmid preparation. A common solution to overcome these issues is to generate multiple plasmids containing shorter sequences encompassing the complete viral genome [8,9]. However, to obtain a full-length DNA genome, all plasmids need to be assembled by in vitro ligation prior to RNA synthesis, which reduces the overall efficiency of the in vitro transcription reaction. This can then result in low yields of RNA transcripts and low transfection efficiency with poor recovery of infectious viruses. 

Thus, the availability of a full-length genome DNA clone would be highly advantageous as it simplifies the production of in vitro transcripts for efficient transfection and rescue of viable viruses, and could be achieved by using a bacterial artificial chromosome (BAC) [10,11]. The BAC is a low-copy number plasmid, which allows the introduction of large DNA sequences in bacteria while minimizing their instability. In addition, the BAC is easy to manipulate in bacteria like other conventional plasmids. Although yeast artificial chromosomes (YAC) have also been used for constructing large DNA clones [12], BAC systems are usually preferred and have been successfully used to construct coronavirus infectious clones, including clones for SARS-CoV and recently SARS-CoV-2 [10,11,13,14,15,16]. 

Remdesivir was the first direct-acting antiviral to be approved for the treatment of COVID-19, and has been recently joined by molnupiravir, another polymerase inhibitor, in the fight against SARS-CoV-2 infection [17,18]. However, research is needed to assess the impact of SARS-CoV-2 mutations on drug susceptibility [19,20]. 

Here, we constructed a full-length SARS-CoV-2 clone using a BAC system. In addition, we introduced luciferase and green florescence protein (GFP) genes into the clone to generate SARS-CoV-2 high-throughput reporter systems. Using the developed systems, we investigated drug susceptibility of selected nsp12 polymerase mutants, including mutations shown to confer remdesivir resistance in other coronaviruses, compared to the original isolate. Finally, we generated highly replicative but non-infectious SARS-CoV-2 clones by deleting selected structural proteins.

## 2. Materials and Methods

### 2.1. Generation of a Full-Length SARS-CoV-2 cDNA by RT-PCR 

The clone is based on isolate SARS-CoV-2/human/Denmark/DK-AHH1/2020 (DK-AHH1, GenBank accession number MZ049597), which was obtained from the nasopharyngeal swab of a SARS-CoV-2 infected individual. Extraction of viral RNA from the clinical material was done as previously described [21]. Subsequently, cDNA was generated in four individual RT reactions with primers RT1 (reaction 1), RT2 (reaction 2), RT3 (reaction 3), and anchored oligo dT_20_ (reaction 4) using previously described conditions [22]. The sequences of all primers used in this study can be found in Table 1.

The cDNA fragments were then amplified in four PCR reactions (excluding the PolyA tail) with primers PF1 and PR1 (reaction 1), PF2 and PR2 (reaction 2), PF3 and PR3 (reaction 3), and PF4 and PR4 (reaction 4) (Table 1). Cycling conditions were 30 s at 98 °C followed by 35 cycles of 98 °C for 10 s, 65 °C for 10 s, 72 °C for 8 min, and a final step of 72 °C for 8 min. The PCR products were verified on a 1% agarose gel, extracted using the Zymo Large fragment kit (ZymoResearch, Irvine, CA, USA), cloned using the TOPO-XL2 kit (Invitrogen, Waltham, MA, USA) and transformed in *E. coli* One Shot OmniMAX 2 T1^R^ (ThermoFisher, Waltham, MA, USA). Subsequently, insertions were verified by Sanger sequencing using the Sequencher v. 5.3 software (Gene Codes, Ann Arbor, MI, USA). 

Sequence toxicity was observed when cloning PCR product 3 (reaction 3). To overcome toxicity problems, we cloned the fragment into the pBeloBAC11 vector using the InFusion cloning kit (Takara, Kusatsu, Shiga, Japan) and transformed into DH10B cells (ThermoFisher, Waltham, MA, USA). Site-directed mutagenesis was performed on fragment 3 to remove one non-synonymous mutation using the mega primer approach, using an in-house developed method as previously described [23].

### 2.2. Construction of a Full-Length SARS-CoV-2 BAC Clone

In order to assemble all genetic elements, we first modified the four PCR fragments to add a 15 bp overlap between them with primers found in Table 1. For fragment 1, the forward primer (F1-T7) contained a T7 promotor sequence upstream of the first nucleotide in the viral genome. Similarly, fragment 4 was modified by adding a polyA tail and a 15-bp overlap with the hepatitis delta virus (HDV) ribozyme sequence (F4-A-RZ). The HDV-ribozyme sequence was amplified with primers RZ-F and RZ-R from a plasmid (a gift from V. Lohmann). 

The BAC vector was then linearized by PCR using primers BAC11-RZ-F and BAC11-F4-R that introduced overlaps with fragment 4 and the HDV-ribozyme sequence. PCR products were DpnI-treated and purified by gel extraction for subsequent stepwise assembly. In step 1, fragment 4 was fused to the HDV-ribozyme and the BAC vector using the InFusion kit (Takara, Kusatsu, Shiga, Japan) for cloning. DH10B bacteria (10-beta, New England Biolabs, Ipswich, MA, USA) were used for transformation and clones were screened by both restriction enzyme digestion with EcoRI and with Sanger sequencing. In step 2, positive clones from step 1 were linearized by PCR using primers that introduced a 15-bp overlap with fragment 2 (F4-F and Bac11-F2-R), followed by infusion cloning. In step 3, recovered and verified clones from step 2 were linearized by PCR, which introduced overlaps with fragment 1 in each end (F2-F and BAC11-T7-R). This allowed for the final InFusion cloning. 

The final full-length BAC clone contains a T7 promoter at the 5′-end, followed by the complete virus sequence, a polyA tail of 33 A’s, the HDV ribozyme sequence, and a NotI restriction site at the 3′ end of the virus sequence. The final plasmid was prepared using QIA Spin miniprep or large-construct kits (Qiagen, Hilden, Germany) and sequence-confirmed by next-generation sequencing (NGS). 

### 2.3. Modifications of the Full-Length BAC Clone: Mutagenesis, Insertions, and Deletions 

Point mutations in nsp12 (L323P, A97V, N491S, F480L, and V557L) were introduced using an in-house mega primer PCR approach as previously described [23] with minor modifications (35 min elongation time instead of 20 min).

Insertion of di-nucleotide-optimized green florescent protein (GFP) [24], di-nucleotide-optimized firefly luciferase (Fluc) [24], and nanoluciferase (nLuc) reporter genes and generation of gene deletions were all performed by InFusion cloning (Takara, Kusatsu, Shiga, Japan). The reporter genes were inserted into ORF7 either replacing the entire region or leaving 36 bp upstream and 182 bp downstream of the ORF7 gene [9]. An extra stop codon was inserted after the end of the reporter to avoid C-terminal amino acids additions. 

To introduce deletions, we designed primers with 15 bp overlaps spanning the introduced gap to allow the InFusion reaction specifically to reconnect the ends thereby generating the desired deletions. The final plasmids were prepared using QIA Spin miniprep or large-construct kits (Qiagen, Hilden, Germany) and were sequence-confirmed by NGS. 

### 2.4. Virus Cell Culture

All cell culture experiments were performed in African green Monkey (Vero E6) cells, as described previously [22]. Briefly, Vero E6 cells were cultured in Dulbecco’s modified Eagle’s medium (DMEM) (Invitrogen, Waltham, MA, USA) supplemented with 10% fetal bovine serum (FBS; Sigma) and antibiotic-antimycotic (100 U/mL of penicillin, 100 μg/mL of streptomycin, and 0.25 μg/mL of amphotericin B; Gibco) and kept at 37 °C in a humidified incubator. 

Infectivity titers were calculated as 50% tissue culture infectious doses per milliliter (TCID_50_/mL). Briefly, cells were infected in 96-well plates with 100 μL of serially diluted supernatants, in quadruplicates, and further processed for immunostaining at 72 h post-infection using SARS-CoV-2 spike chimeric monoclonal primary antibody (40150-D004, Sino Biological, Beijing, China) and anti-human secondary antibody conjugated to horseradish peroxidase (A24476, Invitrogen, Waltham, MA, USA), as previously described [22]. Presence of infection in each replicate was evaluated and used to determine TCID_50_/mL based on the Reed and Muench method [25]. 

Experimental procedures for remdesivir treatments have been described in detail previously [22]. Briefly, cells were seeded in 96-well plates and 24 h afterwards remdesivir at different concentrations and viral supernatants containing viruses were added simultaneously, followed by incubation for 72 h. Plates were subsequently immunostained as described above and infected cells were counted. The 50% effective concentration (EC_50_) values were calculated by nonlinear regression analysis. 

### 2.5. Transfections and Luciferase Assay

For full-length clones, transfection experiments were carried out in 12-well plates in which 100,000–200,000 cells per well were seeded 1–2 days prior to transfection. 2–5 µg of plasmid DNA was digested with NotI for linearization. The linearized plasmid was then purified with the Zymo DNA clean&concentrator-25 kit but using Zymo-Spin™ IC-XL columns (ZR BAC DNA Miniprep Kit, ZymoResearch, Irvine, CA, USA) to efficiently capture the large plasmid. 0.5–1 µg of linearized plasmid was used for in vitro transcription using the mMESSAGE mMACHINE T7 Transcription Kit (ThermoFisher, Waltham, MA, USA) and following the manufacturer’s instructions. The RNA transcripts were quantified using the Qubit RNA BR Assay Kit (ThermoFisher, Waltham, MA, USA) and were then used for transfection.

Transfection was performed with lipofectamine 2000, by diluting 0.5–2 μg of RNA in 250 µL of Opti-MEM (Invitrogen, Waltham, MA, USA) and mixing with 5 µL of lipofectamine also diluted in Opti-MEM to a final volume of 250 µL. The complexes were incubated for 20 min at room temperature and added to cells that were pre-washed with PBS and contained 500 µL of Opti-MEM, thus the final transfection reaction volume was 1 mL. Transfected cells were incubated for 3–4 h, and the transfection reaction was replaced with fresh media. Evaluation of virus induced cytopathic effect (CPE) after transfection was performed by visual inspection of the cells with an inverted light microscope. 

Fluc and nLuc activity were measured using the Luciferase Assay System and Nano-Glo^®^ Luciferase Assay System (Promega, Madison, WI, USA), respectively, following the manufacturer’s instructions. Luciferase activity was detected with a Synergy LX Multi-Mode Microplate Reader (Biotek, Winooski, VT, USA) and expressed as relative light units (RLU).

Transfections of nLuc reporter clones with deletions in structural genes were performed in 12-well plates seeded with 200,000 cells per well 24 h prior. Transfection reactions were prepared as described above with 0.5, 1, or 2 µg of RNA in triplicates for each measured time point. Mock transfections were performed without RNA input. Transfection reactions were removed from the cells after 1 or 4 h depending on the experiment and replaced with fresh media, and reporter activity was measured in the cell lysate for the first time point (1 or 4 h). For subsequent time points, supernatants were harvested, and luciferase activity was measured as described above. 250 µL of supernatant recovered from the 72-h time point was used to inoculate naïve (never infected) Vero E6 cells to confirm that clones were non-infectious. Inoculated cultures were kept for 15 days and the absence of CPE at any time point was confirmed by visual inspection of the cultures. In dose response assays with the nLuc construct, remdesivir was added 32 h prior to transfection and again at 1-h post transfection.

### 2.6. Microscopy and Image Analysis

Vero cells were infected with indicated viruses (MOI 0.01), plated on chamber slides, and fixed at specific time points in 4% formaldehyde for 30 min. Fixed slides were washed with PBS-Tween 0.1% followed by virus-specific immunostaining with primary antibody diluted in PBS containing 0.5% saponin, 1% Glycine, 1% BSA, and 0.2% skimmed milk overnight at 4°C. Primary antibodies were human anti-SARS-CoV-2 Spike S1 Antibody (40150-D004; Sino Biologicals, Beijing, China) and rabbit anti-SARS-CoV-2 Nucleocapsid (40143-R001; Sino Biologicals, Beijing, China). Secondary antibody incubation was performed in PBS containing 1% BSA and 0.2% skimmed milk for 1 h at room temperature with AlexaFluor555 anti-human, AlexaFluor647 anti-rabbit, and Hoechst 33342 (ThermoFisher, Waltham, MA, USA). After washing, cells were mounted using Prolong Glass (ThermoFisher, Waltham, MA, USA) and cured for 24 h. Imaging was performed on a Zeiss AxioObserver Z1 using a 40× NA 1.3 objective, and an Andor iXon 897 Camera at 512 × 512 scanning resolution. Image acquisition included four channels: Hoechst, GFP, AlexaFluor 555, and AlexaFluor 647. Image analysis and post-processing was performed using Zen version 3.2 (Zeiss, Oberkoche, Germany), and image composition was done in Illustrator version 24 (Adobe, San Jose, CA, USA). 

### 2.7. Next Generation Sequencing of Viral Supernatants and Plasmids

For viruses, RNA extraction and amplification of nearly full-length genomes using 5 overlapping RT-PCRs were performed as described previously [22]. The DNA from the five PCR reactions was purified using the Zymo DNA clean&concentrator-25 kit and pooled in equal amounts.

Library preps for Illumina sequencing were prepared using the NEBnext ultra II FS DNA kit (New England Biolabs, Ipswich, MA, USA). For both PCR pools and plasmids, 25 ng of DNA were used as input. Library preps produced fragments of 500–600 bp and were sequenced on a Miseq using the 250 bp pair-end setting with the v2 500 cycles sequencing kits (Illumina, San Diego, CA, United States). Sequence analysis was performed as described previously for rescued viruses [26] but using Cutadapt [27] to trim off internal primer sequences located at the 5′-end of the read. Plasmid construct sequences were confirmed in the Geneious Prime software (Auckland, New Zealand).

### 2.8. RT-qPCR

RT-qPCR was performed using protocols described previously [28] and the reaction setup was adapted to TaqMan™ Fast Virus 1-Step Master Mix (ThermoFisher, Waltham, MA, USA). RNA extracted for sequencing as described above was diluted 100-fold and assayed using the E nucleotide sequence as a target for primers and probe. The assay was run on a Lightycler 96 (Roche, Basel, Switzerland) and analyzed with the Lightcycler 96 software (Roche, Basel, Switzerland) version 1.10.1320. SARS-CoV-2 genome equivalents per mL (GE/mL) were calculated by interpolation in an in-run standard curve based on synthetic SARS-CoV-2 RNA controls (Twist Bioscience, South San Francisco, CA, USA) [29]. 

## 3. Results

### 3.1. Characterization of a Novel SARS-CoV-2 Full-Length Clone 

**Figure 1:**
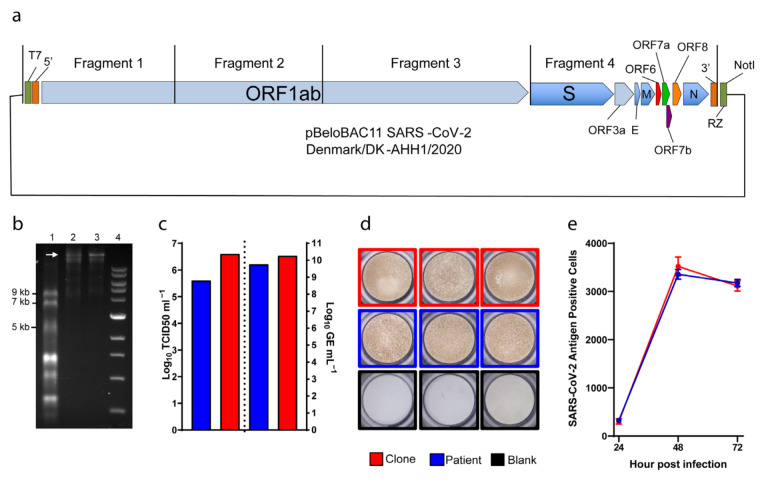
Reverse-genetics system for SARS-CoV-2. (**a**) Diagram of the bacterial artificial chromosome (BAC), in which the complete genome (from four cloned fragments as indicated) of SARS-CoV-2 was inserted. Individual open reading frames (ORFs) are depicted as arrow boxes. 5′ and 3′: untranslated regions; RZ: HDV-ribozyme; NotI: restriction site. (**b**) In vitro transcription of the full-length SARS-CoV-2 plasmid, 40 ng of RNA transcript loaded per well. 1: ssRNA ladder; 2: DNase-treated RNA transcripts; 3: RNA transcripts; 4: 1kb DNA ladder. The white arrow indicates full-length RNA transcripts. (**c**) to the left of the dotted line, representative peak infectivity titers (TCID_50_/mL) of the second passage virus. To the right, RNA titers (as GE/mL) of the same samples measured by E-sequence specific RT-qPCR. (**d**) Representative pictures of infected immunostained Vero E6 cells. (**e**) Virus propagation kinetics after infection at MOI 0.01. Graphs indicate the number of infected cells determined by immunostaining (*y*-axis) at different time points (*x*-axis). Data are shown as mean and standard error of the mean. Patient (blue): virus isolated from a COVID-19 patient [22]; clone (red): virus rescued from RNA transcribed from the clone illustrated in (**a**); blank (black): culture medium.

A SARS-CoV-2 full-length BAC clone (Figure 1a), representing the consensus genome sequence of isolate DK-AHH1 [22], was constructed using InFusion cloning technology as detailed in the Materials and Methods. 

In vitro transcription and capping of 0.5–1 μg of linearized BAC clone produced full-length SARS-CoV-2 RNA with yields of over 40 μg per reaction, and the size of RNA transcripts could be confirmed by running the reaction products on a formaldehyde-containing agarose gel (Figure 1b). 

To verify viability of the viral clone, in vitro-transcribed RNA was transfected into Vero E6 cells and infection spread, monitored by the detection of CPE, could be observed already at day 1 after transfection. Supernatants from transfected cultures were used to infect naïve Vero E6 cells and CPE was observed at day 3 after infection. A second passage was performed, and CPE was observed at day 2 post-infection. These first results suggested that the clone was viable as it was capable of efficiently infecting and replicating in Vero E6 cells.

To further assess viability of our clone, we determined peak infectivity and RNA titers of second passage viral supernatants and of the original viral isolate grown in Vero E6 cells in similar conditions (referred to as patient) (Figure 1c). Viability of the clone and patient viruses was comparable, with the clone spreading slightly faster and reaching higher infectivity (6.6 Log versus 5.6 Log TCID_50_/mL, respectively) and RNA titers (10.3 Log versus 9.8 Log GE/mL, respectively). Furthermore, cells infected by either clone or patient virus could be similarly immunostained using anti-SARS-CoV-2 spike antibody (Figure 1d). Finally, we performed a three-day infection experiment to verify the viral spread kinetics of the clone and patient viruses. Vero E6 cells were infected at MOI of 0.01 and monitored every 24 h for viral infection using immunostaining (Figure 1e). Both viruses spread similarly as shown by the increase in the number of infected cells over time, suggesting comparable growth kinetics. Overall, this data showed that our clone could recapitulate the phenotype of the original patient isolate in cell culture. 

Viral RNA obtained from second passage supernatants of the clone culture was sequenced by NGS to confirm its genetic stability. We could not detect any amino acid (aa) changes in the consensus sequence (at least 50% of population) of the recovered viruses, indicating that the original sequence of the clone led to efficient virus growth in cell culture. However, as previously demonstrated [22], the virus evolves in cell culture and a single change in the S coding region (S247R) was already detected in over 40% of the viral population (Table 2).

### 3.2. Generation of Marker SARS-CoV-2 Viruses 

The ORF7 region of SARS-CoV-2 has previously been shown to be permissive to the insertion of reporter genes and was therefore chosen for insertion of GFP, Fluc, and nLuc reporter genes [9,30]. Initially, reporter genes were inserted into ORF7a leaving the 5′- and 3′-ends of the original sequence, including the start and stop codons, using the InFusion kit. To assess viability and genetic stability of the constructs, in vitro transcribed RNA was transfected into Vero E6 cells and produced viruses were subsequently passaged twice into naïve cells. 

**Figure 2:**
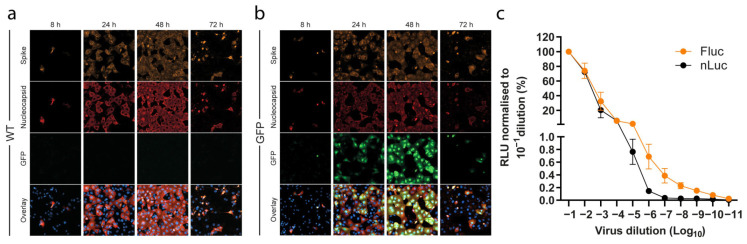
Clone reporter systems for SARS-CoV-2. (a,b) Representative images of SARS-CoV-2 Spike, SARS-CoV-2 Nucleocapsid, and GFP channels, together with an overlay of all channels including the nucleus, from a time-course infection experiment (MOI 0.01) of (a) wild-type clone (WT) and (b) GFP-reporter viruses, taken with a 40× objective. (c) Titration of nLuc and Fluc reporter activity of SARS-CoV-2 reporter viruses with initial infectivity titers of 5.5 and 5.3 Log TCID_50_/mL, respectively. The values of relative light units (RLU) measured at 24 h were subtracted from negative control wells and normalized to the values obtained from the 10^−1^ virus dilution. The data points represent mean of quadruplicates; error bars represent standard error of the mean. For some data points the differences are too small to plot error bars.

Sequence analysis of viruses recovered after transfection and second passage infections confirmed that constructs had maintained the reporter GFP and nLuc, but not the Fluc inserted sequences. Time-course infection experiments with the SARS-CoV-2-GFP second passage virus showed fluorescence signal that could be detected already 8 h post infection and that co-localized with both S and N viral proteins marked by immunofluorescence (Figure 2a,b). The number of GFP positive cells increased until 48 h post infection, but the fluorescence signal declined at 72 h, likely due to CPE and the corresponding loss of cells (Figure 2a,b). The expression of N and S viral proteins followed a similar pattern. To verify the functionality of the nLuc construct, we performed a luciferase titration assay using the Nano-Glo kit (Figure 2c). Vero E6 cells were infected in triplicate with a serial dilution of the second passage nLuc virus and incubated for 24 h. Subsequent quantification of luciferase signal showed that the nLuc virus produced strong dose-dependent luciferase signal. These results indicated that both the GFP and the nLuc reporter-gene constructs produce a strong, reliable signal, suitable to high-throughput assays. 

A second attempt at obtaining a genetically stable Fluc virus was performed by inserting the Fluc gene in place of the entire ORF7, leaving no original sequence. This resulted in a virus that maintained the reporter sequence after second passage. Further, the Fluc clone viruses yielded measurable luciferase signals after 24 h in a dose-dependent manner, similar to the nLuc construct (Figure 2c). Attempts at applying the same cloning strategy to the nLuc and GFP reporters resulted in unstable constructs and low level of reporter gene activity, respectively. 

### 3.3. Suitability of Full-Length SARS-CoV-2 Clones for Drug Susceptibility Testing 

**Figure 3:**
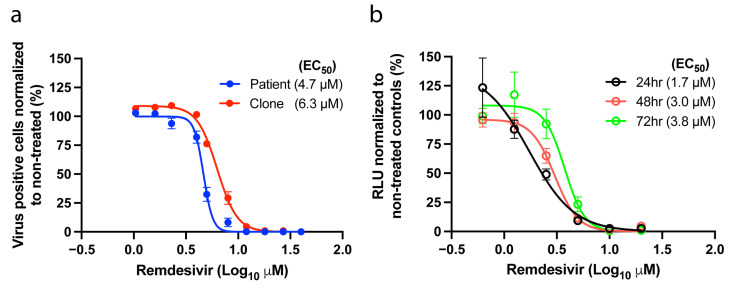
Susceptibility to remdesivir. (a) Antiviral activity of remdesivir against the patient and the clone viruses (passage 2 viruses). (b) Antiviral potency of remdesivir against the nLuc reporter virus measured 24, 48, and 72 h after treatment using a luciferase assay. The graphs show the non-linear regression curve of the number of SARS-CoV-2 infected cells treated with different remdesivir concentrations normalized to non-treated controls. The dots and circles represent the mean of triplicates; error bars represent standard error of the mean. For some data points the differences are too small to plot error bars. EC_50_ values (µM) inferred from the regression are shown in parenthesis.

To assess whether our novel SARS-CoV-2 constructs could be used in drug susceptibility assays, we first performed drug concentration–response assays applying remdesivir to Vero E6 cells infected with either the full-length clone or the original patient isolate (Figure 3a). In this short-term assay both viruses showed similar susceptibility to remdesivir, resulting in effective concentration 50% (EC_50_) values of 6.3 µM and 4.7 µM for the clone and the patient virus, respectively (Figure 3a).

We next validated the nLuc reporter virus for the testing of antiviral compounds, using remdesivir as a proof-of-concept. In a concentration-response assay, the reporter virus was inhibited in a dose-dependent manner as early as 24 h post-treatment. EC_50_ values at 48 and 72 h were comparable (within 2-fold differences) to the values obtained in antigen-based treatment assays of the patient and clone viruses, making this a valuable tool for the rapid and high-throughput screening of drug candidates (Figure 3b). 

### 3.4. Remdesivir Susceptibility of SARS-CoV-2 nsp12 Mutant Viruses 

To address whether naturally occurring nsp12 polymorphisms and previously described nsp12 coronavirus remdesivir resistance mutations [19] influence SARS-CoV-2 drug susceptibility, we introduced selected mutations into the full-length clone and performed remdesivir concentration–response assays with each mutant.

Among natural nsp12 polymorphisms, we investigated the P323L mutation, known as the major polymorphic site of nsp12 (>90% prevalence found in GISAID EpiCov database accessed 10 May 2021). Our patient-isolate already carried an L in contrast to the Wuhan-1 reference sequence (NC_045512), which carried a P, thus we generated the L323P mutant. Additionally, we investigated two low prevalence variants found in the GenBank database, A97V, and N491S.

To verify whether the nsp12 mutations F480L and V557L conferring SARS-CoV resistance to remdesivir (6-fold increase in EC_50_) in cell culture [19], would in fact also confer SARS-CoV-2 resistance, we engineered these mutations into our full-length clone both individually and in combination. 

**Figure 4:**
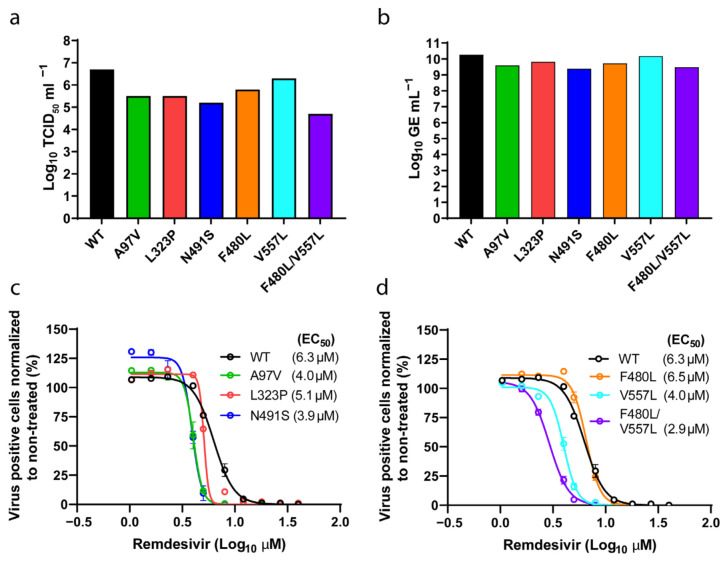
Analysis of SARS-CoV-2 nsp12 polymerase mutants. (a) Peak infectivity titers (TCID_50_/mL) of second passage SARS-CoV-2 viruses with specified nsp12 substitutions and of the parental wild-type clone (WT). (b) SARS-CoV-2 RNA titers of the same samples measured by E-sequence specific RT-qPCR. (c,d) Concentration–response curves of infections from the constructs depicted in (a) and (b) treated with different remdesivir concentrations. The dots represent the mean, and standard errors of the mean are shown as lines. EC_50_ values (µM) inferred from the regression analysis are shown in parenthesis for each construct. For some data points the differences are too small to plot error bars.

All generated mutants were viable in cell culture exhibiting peak infectivity titers above 4 Log TCID_50_/mL and corresponding high RNA titers above 9 Log GE/mL (Figure 4a,b). All mutants maintained the engineered mutations up to second passage and did not acquire additional mutations in nsp12 (Table 2).

The remdesivir susceptibility of the mutants was then assessed in a short-term concentration–response assay. All SARS-CoV-2 nsp12 mutants were similarly inhibited by remdesivir and showed EC_50_s ranging from 2.9 µM to 6.5 µM, which were comparable to the unmodified clone (6.3 µM) and to the patient virus (4.7 µM) (Figure 3a and Figure 4c,d). These results indicate that neither the identified nsp12 polymorphic changes nor the previously described coronavirus resistance-associated substitutions V557L and F480L significantly affect SARS-CoV-2 drug susceptibility under our experimental conditions. 

### 3.5. Non-Infectious Subgenomic SARS-CoV-2 Clones with Efficient RNA Replication Inhibited by Remdesivir 

**Figure 5:**
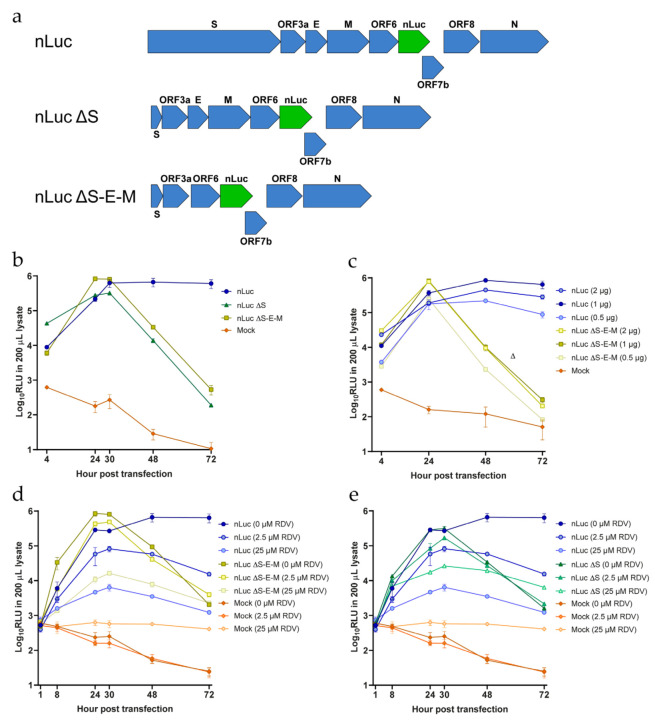
Generation of SARS-CoV-2 subgenomic replicon systems. (a) Structure of the replicon constructs, with the nLuc ORF7a insertion highlighted in green. (b) Luciferase activity of the different replicons after transfection with 2 μg of RNA. (c) Luciferase activity after transfection with different amounts of RNA transcripts. (d,e) Luciferase activity of the different SARS-CoV-2 nLuc systems under treatment with remdesivir (RDV) at various concentrations. Remdesivir was added 32 h prior to transfections and re-applied 1-h post transfection. Mock, transfection without any RNAs. The datapoints represent the mean of three independent transfections. Each transfection was performed in triplicate, and the mean of each transfection was used to calculate the values shown here; error bars represent standard errors of the mean. For some data points the differences are too small to plot error bars.

To investigate whether it was possible to develop an efficient t7-BAC subgenomic non-infectious RNA-replication system for SARS-CoV-2, we deleted either the S protein alone or the S, E, and M proteins together from the SARS-CoV-2-nLuc reporter clone described above. To produce the ΔS mutant, we deleted most of the S coding region, leaving 30 nt of the N-terminus and 105 nt of the C-terminus from the original sequence (Figure 5a). In addition, we mutated the start codon and the TRS upstream of the S ORF to further disrupt the S reading frame. The ΔS-E-M mutant was produced by removing the sequences encoding the E and M proteins from the ΔS clone, thus obtaining a construct lacking all three structural proteins (Figure 5a). 

We then assessed the replication capacity of both constructs by transfecting Vero E6 cells with in vitro transcribed RNA. Production of viral RNA was then monitored indirectly by measuring luciferase production over time. The nLuc signal increased (up to 100-fold) from 4 to 24 h (Figure 5b) for both the ΔS and ΔS-E-M clones. The luciferase signal peaked between 24 and 30 h, decreased sharply at 48 h and was almost undetectable at 72 h, suggesting transient replication. In contrast, the luciferase signal of the full-length nLuc virus was maintained at 48 and 72 h, indicating viral propagation (Figure 5b). Neither ΔS nor ΔS-E-M transfected cultures showed any signs of CPE up to 72 h post transfection. To confirm that our subgenomic constructs were non-infectious, we used supernatants harvested from ΔS and ΔS-E-M cultures at 72 h post-transfection to inoculate naïve Vero E6. Cells were monitored every 2–3 days for 15 days after infection and neither CPE nor SARS-CoV-2 N-protein detected by immunofluorescence could be observed. 

We also tested whether differences in the input RNA amount used in the transfection affected the signal detected and we observed that 1 μg of RNA was optimal as higher amounts of RNA neither increased the signal nor prolonged it (Figure 5c). 

Finally, we tested if the observed replication activity could be inhibited by remdesivir. We used remdesivir at low (2.5 µM, equivalent to the EC_50_) and at high (25 µM, fully suppressive) concentrations according to experiments conducted with the full-length clone. Here we observed a clear concentration-dependent inhibition of replication for all three constructs with the high concentration decreasing replication more than 100-fold, although not completely suppressing it (Figure 5d,e). Furthermore, the low dose treatments resulted in intermediate inhibition with 5–10-fold reduction of replication.

## 4. Discussion

Highly versatile reverse-genetics systems for SARS-CoV-2 such as the ones developed in this work are valuable tools for the study of emerging mutations. There are pressing concerns for the emergence of new variants as the SARS-CoV-2 pandemic evolves. Spread of new variants with resistance or evasion mutations could potentially affect drug susceptibility and immune responses. Our systems represent optimal platforms for the characterization of emerging variants harboring mutations in any genomic region. Reverse-genetics systems are also a key tool for the study of the viral life cycle and the importance of genomic regions for virus viability and pathogenicity. In addition, subgenomic reporter replicon systems greatly facilitate studies of viral replication and high throughput screening of compounds inhibiting replication, accelerating the process of antiviral drug discovery. 

Several SARS-CoV-2 reverse genetics systems are currently available, including BAC systems [8,9,11,12]. Compared to those systems, which use a CMV promoter, we here used a T7 promoter as previously described by He et al. [31], thereby allowing in vitro transcription directly from the linearized plasmid. This approach allows for an equally fast turnover as other BAC based methodologies (3–5 day workflow) and for reliable transient assays as the viral genome is not continually transcribed in the transfected cells as is the case with ‘always on’ promoters. Additionally, RNA yields are consistently in the tens of micrograms quantity allowing for a reduction in the number of experimental steps when compared to in vitro ligation systems and thus increasing consistency between input-sensitive experimental setups such as those required for viral evolutionary dynamics experiments.

Our GFP, Fluc, and nLuc reporter systems proved efficient and could be used for the high-throughput screening of drugs, as shown for remdesivir, obtaining EC_50_ values that are in agreement with other studies [8,9,32]. Surprisingly, the Fluc reporter was only functional when the entire ORF7 was removed, suggesting that the specific deletion and the reporter inserted were critical for reporter function and stability. Indeed, inserting Fluc into ORF7a led to rapid reversion and deletion of most of the Fluc insert. GFP was maintained but not expressed when replacing the entire ORF7 with GFP, and only the ORF7a replacement with GFP allowed expression, while nLuc was attenuated when it was inserted, replacing the entire ORF7. Whether this was due to RNA structure in the TRS region, sequence recognition, or other mechanisms requires further studies.

Remdesivir, approved for treatment of SARS-CoV-2 infected patients in many countries, is a potent antiviral targeting the coronavirus RNA dependent RNA polymerase (nsp12) [17]. Therefore, it is relevant to determine if mutations in nsp12 could affect drug susceptibility. The most common naturally occurring polymorphism found in the nsp12 protein, Leucine at position 323 is located at the interface domain of nsp12. Our clone already harbors Leucine compared to the Proline found in the Wuhan reference sequence which was described early during the pandemic [33], and we therefore tested the L323P mutant to confirm remdesivir response was comparable between both variants. In addition to position 323, we investigated two other nsp12 polymorphisms representing minor circulating variants, A97V situated in the N-terminal and N491S found in the fingers subdomain of the polymerase domain. None of these substitutions decreased remdesivir susceptibility in short-term treatment assays, suggesting that these naturally found polymorphisms would not compromise the efficacy of remdesivir treatments [34]. For SARS-CoV, substitutions F480L and V557L (located in the fingers subdomain of the polymerase), were found to be associated with resistance to remdesivir [19]. In our study of SARS-CoV-2, these mutations were tolerated and did not revert, but the V557L mutant spread slower and acquired multiple additional mutations in spike after second passage, suggesting that this mutation had a negative impact on fitness. Interestingly, none of the studied mutations affected remdesivir susceptibility, although mutations at position 557 have been associated with remdesivir resistance in coronaviruses and Ebola virus [19,35,36]. Further studies will be needed to determine, which mutations are involved in remdesivir resistance for SARS-CoV-2, and our high-throughput and versatile reverse-genetics system can easily be used for this purpose.

Subgenomic non-infectious replicative clones (replicons) allow rapid testing of drugs inhibiting replication and the study of the viral replication cycle in lower biosafety-grade laboratories thereby making SARS-CoV-2 research faster, more accessible, and more affordable. Two previous studies on SARS-CoV-2 used the ligation approach with deletion of all structural ORFs from S to ORF8 to create such systems [6,37]. For other coronaviruses including SARS-CoV, a similar approach was successfully used to construct and launch replicons [13,38,39]. Our approach to generate the subgenomic non-infectious clone was based on the modification of a viable full-length nLuc construct, by removing the structural proteins S, E, and M, a concept that proved functional for SARS-CoV and recently SARS-CoV-2 [7,40,41]. This strategy was successful with both ΔS and ΔS-E-M clones replicating transiently but efficiently. Previous studies of SARS-CoV-2 replicons reported similar replication kinetics [6,7,31,37]. Remdesivir could inhibit replication in a concentration-dependent manner showing the potential of the system for replicase inhibitor screening. Compared to other available replicon systems [6,7,37], our clones conserve all the accessory proteins, enabling studies of their role in replication.

In conclusion, we have developed a highly versatile and robust reverse genetics system for SARS-CoV-2. This led to the establishment of reporter viruses and subgenomic non-infectious replicons that facilitated high-throughput screening of antiviral activity. We demonstrate the convenience and robustness of the system by generating several mutants and studying their sensitivity to remdesivir in vitro. We showed that remdesivir activity is maintained against naturally occurring nsp12 polymorphisms and against reported SARS-CoV resistance mutations. Due to its simplicity and robustness, we believe that this system, together with in vivo animal models, such as the Syrian golden hamster model [42], can contribute to further our understanding of the SARS-CoV-2 biology and expedite translational studies.

## Figures and Tables

**Table 1:** Primers used to amplify and clone SARS-CoV-2.

Primer ID	Sequence 5′-3′
RT1	CTACATAAGCAGCCATTAGATCT
RT2	TTTGTGTAGTACCGGCAGCA
RT3	AGTGTATGCAGGGGGTAATTG
PF1	ATTAAAGGTTTATACCTTCCCAGGTAACAAAC
PR1	TGTGTGGCCAACCTCTTCTGT
PF2	GGGAATGGATAATCTTGCCTGCG
PR2	ACCGGCAGCACAAGACATCT
PF3	AGGGCCAATTCTGCTGTCAAA
PR3	TGCAGGGGGTAATTGAGTTCTGG
PF4	GGCAAACCACGCGAACAAAT
PR4	GTCATTCTCCTAAGAAGCTATTAAAATCACATG
PF3-In	CAATTACCCCCTGCACCGGGCCGTCGACCAATTC
PR3-In	AGCAGAATTGGCCCTATCGAATATAACTTCGTATAATGTATGCTATACG
F1-T7	TAATACGACTCACTATAGATTAAAGGTTTATACCTTCCCAGGTAACAAAC
F4-R-A-RZ	GGGACCATGCCGGCCTTTTTTTTTTTTTTTTTTTTTTTTTTTTTTTTTGTCATTCTCCTAAGAAGCTATTAAAATCACATG
BAC11-RZ-F	AATGGCGAATGGGACGCGGCCGCCCGGGCCGTCGA
BAC11-F4-R	TGTTCGTTTAGTTGTATCGAATATAACTTCGTATAATGTATGCTATACG
BAC11-F2-R	AGATTATCCATTCCCATCGAATATAACTTCGTATAATGTATGCTATACG
F4-F	ACAACTAAACGAACAATGTTTGTTTTTCTTG
BAC11-T7-R	TAGTGAGTCGTATTAATCGAATATAACTTCGTATAATGTATGCTATACG
F2-F	GGGAATGGATAATCTTGCCTGCG
RZ-F	GGCCGGCATGGTCCCAGCCT
RZ-R	GTCCCATTCGCCATTACCGAG

**Table 2:** Sequence of the SARS-CoV-2 viruses recovered in second passage in Vero E6 cells. The full-length coding regions of the viruses were obtained by NGS. Only non-synonymous SNPs with frequencies >20% are shown. The sequences were compared to Wuhan reference sequence (NC_045512) but only the polymorphism P323L is reported in the table.

Position	Protein	Reference Base	Alternative Base	Patient	WT Clone	nsp12 L323P	nsp12 A97V	nsp12 N491S	nsp12 F480L	nsp12 V557L	nsp12 F480L/V557L	Amino Acid Change ^#^
1,597	nsp2	A	T	-	-	-	-	-	-	43.7	-	E264D
1,877	nsp2	T	A	-	-	-	-	-	-	-	55.5	S358T
2,337	nsp2	A	C	-	-	-	-	59.2	-	-	-	D511A
3,790	nsp3	A	T	-	-	99.5	-	-	-	-	-	L357F
6,063	nsp3	A	G	-	-	39.3	-	-	-	-	-	D1115G
11,075	nsp6	T	C	-	-	-	-	-	20.8	-	-	F35L
11,186	nsp6	T	G	-	-	-	-	-	-	41.2	-	L72V
13,730	nsp12	C	T	-	-	-	99.2	-	-	-	-	A97V
14,408	nsp12	C	T	99.8	99.7	-	99.4	99.8	99.7	99.8	99.7	P323L
14,878	nsp12	T	C	-	-	-	-	-	99.6	-	99.7	F480L
14,880	nsp12	T	A	-	-	-	-	-	99.8	-	99.8	F480L
14,912	nsp12	A	G	-	-	-	-	99.6	-	-	-	N491S
15,109	nsp12	G	C	-	-	-	-	-	-	99.2	99.1	V557L
21,784	S	T	A	-	-	-	-	-	21.0	-	0.7	N74K
22,301	S	A	C	-	42.7	0.8	-	-	-	-	-	S247R
23,525	S	C	T	-	0.3	-	8.5	4.7	-	-	-	H655Y
23,606	S	C	T	-	3.1	33.5	17.5	1.3	-	-	-	R682W
23,615	S	C	A	-	-	-	-	-	99.4	-	-	R685S
26,261	E	C	T	-	1.7	-	45.1	8.0	-	2.5	-	S6L
26,333	E	C	T	-	-	-	0.5	66.6	-	-	1.0	T30I
26,435	E	A	C	-	-	-	-	-	-	81.0	-	N64T
27,224	ORF6	A	C	-	-	-	-	-	-	-	94.3	Q8P

WT: wild-type. #: numbers refer to protein specific residues. -: not detected. Mutations marked in bold green were engineered. Mutation marked in bold red occurred randomly during cloning, but it was outside the target region of study.

## Data Availability

The data presented in this study are available on request from the corresponding author.

## References

[1] Hu B., Guo H., Zhou P., Shi Z.L. (2021). Characteristics of SARS-CoV-2 and COVID-19. Nat. Rev. Microbiol..

[2] Gorbalenya A.E., Baker S.C., Baric R.S., de Groot R.J., Drosten C., Gulyaeva A.A., Haagmans B.L., Lauber C., Leontovich A.M., Neuman B.W. (2020). The species Severe acute respiratory syndrome-related coronavirus: Classifying 2019-nCoV and naming it SARS-CoV-2. Nat. Microbiol..

[3] Cui J., Li F., Shi Z.L. (2019). Origin and evolution of pathogenic coronaviruses. Nat. Rev. Microbiol..

[4] Zhu N., Zhang D., Wang W., Li X., Yang B., Song J., Zhao X., Huang B., Shi W., Lu R. (2020). A Novel Coronavirus from Patients with Pneumonia in China, 2019. N. Engl. J. Med..

[5] V’kovski P., Kratzel A., Steiner S., Stalder H., Thiel V. (2021). Coronavirus biology and replication: Implications for SARS-CoV-2. Nat. Rev. Microbiol..

[6] Xia H., Cao Z., Xie X., Zhang X., Chen J.Y.C., Wang H., Menachery V.D., Rajsbaum R., Shi P.Y. (2020). Evasion of Type I Interferon by SARS-CoV-2. Cell Rep..

[7] Ricardo-Lax I., Luna J.M., Thao T.T.N., Le Pen J., Yu Y., Hoffmann H.-H., Schneider W.M., Razooky B.S., Fernandez-Martinez J., Schmidt F. (2021). Replication and single-cycle delivery of SARS-CoV-2 replicons. Science.

[8] Xie X., Muruato A., Lokugamage K.G., Narayanan K., Zhang X., Zou J., Liu J., Schindewolf C., Bopp N.E., Aguilar P.V. (2020). An Infectious cDNA Clone of SARS-CoV-2. Cell Host Microbe.

[9] Hou Y.J., Okuda K., Edwards C.E., Martinez D.R., Asakura T., Dinnon K.H., Kato T., Lee R.E., Yount B.L., Mascenik T.M. (2020). SARS-CoV-2 Reverse Genetics Reveals a Variable Infection Gradient in the Respiratory Tract. Cell.

[10] Almazán F., González J.M., Pénzes Z., Izeta A., Calvo E., Plana-Durán J., Enjuanes L. (2000). Engineering the largest RNA virus genome as an infectious bacterial artificial chromosome. Proc. Natl. Acad. Sci. USA.

[11] Ye C., Chiem K., Park J.G., Oladunni F., Platt R.N., Anderson T., Almazan F., de la Torre J.C., Martinez-Sobrido L. (2020). Rescue of SARS-CoV-2 from a single bacterial artificial chromosome. MBio.

[12] Thi Nhu Thao T., Labroussaa F., Ebert N., V’kovski P., Stalder H., Portmann J., Kelly J., Steiner S., Holwerda M., Kratzel A. (2020). Rapid reconstruction of SARS-CoV-2 using a synthetic genomics platform. Nature.

[13] Almazán F., DeDiego M.L., Galán C., Escors D., Álvarez E., Ortego J., Sola I., Zuñiga S., Alonso S., Moreno J.L. (2006). Construction of a Severe Acute Respiratory Syndrome Coronavirus Infectious cDNA Clone and a Replicon To Study Coronavirus RNA Synthesis. J. Virol..

[14] Almazán F., Dediego M.L., Sola I., Zuñiga S., Nieto-Torres J.L., Marquez-Jurado S., Andrés G., Enjuanes L. (2013). Engineering a replication-competent, propagation-defective middle east respiratory syndrome coronavirus as a vaccine candidate. MBio.

[15] Rihn S.J., Merits A., Bakshi S., Turnbull M.L., Wickenhagen A., Alexander A.J.T., Baillie C., Brennan B., Brown F., Brunker K. (2021). A plasmid DNA-launched SARS-CoV-2 reverse genetics system and coronavirus toolkit for COVID-19 research. PLoS Biol..

[16] Rathore M.H., Runyon J., Haque T. (2017). Emerging Infectious Diseases. Adv. Pediatr..

[17] Beigel J.H., Tomashek K.M., Dodd L.E., Mehta A.K., Zingman B.S., Kalil A.C., Hohmann E., Chu H.Y., Luetkemeyer A., Kline S. (2020). Remdesivir for the Treatment of Covid-19—Final Report. N. Engl. J. Med..

[18] Mahase E. (2021). Covid-19: Molnupiravir reduces risk of hospital admission or death by 50% in patients at risk, MSD reports. BMJ.

[19] Agostini M.L., Andres E.L., Sims A.C., Graham R.L., Sheahan T.P., Lu X., Smith E.C., Case J.B., Feng J.Y., Jordan R. (2018). Coronavirus Susceptibility to the Antiviral Remdesivir (GS-5734) Is Mediated by the Viral Polymerase and the Proofreading Exoribonuclease. MBio.

[20] Deng X., StJohn S.E., Osswald H.L., O’Brien A., Banach B.S., Sleeman K., Ghosh A.K., Mesecar A.D., Baker S.C. (2014). Coronaviruses resistant to a 3C-like protease inhibitor are attenuated for replication and pathogenesis, revealing a low genetic barrier but high fitness cost of resistance. J. Virol..

[21] Fahnøe U., Bukh J. (2019). Full-length open reading frame amplification of hepatitis C virus. Methods Mol. Biol..

[22] Ramirez S., Fernandez-Antunez C., Galli A., Underwood A., Pham L.V., Ryberg L.A., Feng S., Pedersen M.S., Mikkelsen L.S., Belouzard S. (2021). Overcoming Culture Restriction for SARS-CoV-2 in Human Cells Facilitates the Screening of Compounds Inhibiting Viral Replication. Antimicrob. Agents Chemother..

[23] Fahnøe U., Pedersen A.G., Risager P.C., Nielsen J., Belsham G.J., Höper D., Beer M., Rasmussen T.B. (2014). Rescue of the highly virulent classical swine fever virus strain “Koslov” from cloned cDNA and first insights into genome variations relevant for virulence. Virology.

[24] Wolfisberg R., Holmbeck K., Nielsen L., Kapoor A., Rice C.M., Bukh J., Scheel T.K.H. (2019). Replicons of a Rodent Hepatitis C Model Virus Permit Selection of Highly Permissive Cells. J. Virol..

[25] Reed L.J., Muench H.A. (1938). A simple method of estimating fifty per cent endpoints. Am. J. Epidemiol..

[26] Jensen S.B., Fahnøe U., Pham V.L., Serre S.B.N., Tang Q., Ghanem L., Pedersen M.S., Ramirez S., Humes D., Pihl A.F. (2019). Evolutionary Pathways to Persistence of Highly Fit and Resistant Hepatitis C Virus Protease Inhibitor Escape Variants. Hepatology.

[27] Martin M. (2011). Cutadapt removes adapter sequences from high-throughput sequencing reads. EMBnet.Journal.

[28] Corman V.M., Landt O., Kaiser M., Molenkamp R., Meijer A., Chu D.K.W., Bleicker T., Brünink S., Schneider J., Schmidt M.L. (2020). Detection of 2019 novel coronavirus (2019-nCoV) by real-time RT-PCR. Eurosurveillance.

[29] Offersgaard A., Duarte Hernandez C.R., Pihl A.F., Costa R., Venkatesan N.P., Lin X., Van Pham L., Feng S., Fahnøe U., Scheel T.K. (2021). SARS-CoV-2 Production in a Scalable High Cell Density Bioreactor. Vaccines.

[30] Sims A.C., Baric R.S., Yount B., Burkett S.E., Collins P.L., Pickles R.J. (2005). Severe Acute Respiratory Syndrome Coronavirus Infection of Human Ciliated Airway Epithelia: Role of Ciliated Cells in Viral Spread in the Conducting Airways of the Lungs. J. Virol..

[31] He X., Quan S., Xu M., Rodriguez S., Goh S.L., Wei J., Fridman A., Koeplinger K.A., Carroll S.S., Grobler J.A. (2021). Generation of sars-cov-2 reporter replicon for high-throughput antiviral screening and testing. Proc. Natl. Acad. Sci. USA.

[32] Xie X., Muruato A.E., Zhang X., Lokugamage K.G., Fontes-Garfias C.R., Zou J., Liu J., Ren P., Balakrishnan M., Cihlar T. (2020). A nanoluciferase SARS-CoV-2 for rapid neutralization testing and screening of anti-infective drugs for COVID-19. Nat. Commun..

[33] Pachetti M., Marini B., Benedetti F., Giudici F., Mauro E., Storici P., Masciovecchio C., Angeletti S., Ciccozzi M., Gallo R.C. (2020). Emerging SARS-CoV-2 mutation hot spots include a novel RNA-dependent-RNA polymerase variant. J. Transl. Med..

[34] Martin R., Li J., Parvangada A., Perry J., Cihlar T., Mo H., Porter D., Svarovskaia E. (2021). Genetic conservation of SARS-CoV-2 RNA replication complex in globally circulating isolates and recently emerged variants from humans and minks suggests minimal pre-existing resistance to remdesivir. Antiviral Res..

[35] Lo M.K., Albariño C.G., Perry J.K., Chang S., Tchesnokov E.P., Guerrero L., Chakrabarti A., Shrivastava-Ranjan P., Chatterjee P., McMullan L.K. (2020). Remdesivir targets a structurally analogous region of the Ebola virus and SARS-CoV-2 polymerases. Proc. Natl. Acad. Sci. USA.

[36] Szemiel A.M., Merits A., Orton R.J., MacLean O.A., Pinto R.M., Wickenhagen A., Lieber G., Turnbull M.L., Wang S., Furnon W. (2021). In vitro selection of Remdesivir resistance suggests evolutionary predictability of SARS-CoV-2. PLOS Pathog..

[37] Kotaki T., Xie X., Shi P.Y., Kameoka M. (2021). A PCR amplicon-based SARS-CoV-2 replicon for antiviral evaluation. Sci. Rep..

[38] Ge F., Luo Y., Liew P.X., Hung E. (2007). Derivation of a novel SARS-coronavirus replicon cell line and its application for anti-SARS drug screening. Virology.

[39] Hertzig T., Scandella E., Schelle B., Ziebuhr J., Siddell S.G., Ludewig B., Thiel V. (2004). Rapid identification of coronavirus replicase inhibitors using a selectable replicon RNA. J. Gen. Virol..

[40] Wang J.M., Wang L.F., Shi Z.L. (2008). Construction of a non-infectious SARS coronavirus replicon for application in drug screening and analysis of viral protein function. Biochem. Biophys. Res. Commun..

[41] Zhang Y., Song W., Chen S., Yuan Z., Yi Z. (2021). A bacterial artificial chromosome (BAC)-vectored noninfectious replicon of SARS-CoV-2. Antiviral Res..

[42] Imai M., Iwatsuki-Horimoto K., Hatta M., Loeber S., Halfmann P.J., Nakajima N., Watanabe T., Ujie M., Takahashi K., Ito M. (2020). Syrian hamsters as a small animal model for SARS-CoV-2 infection and countermeasure development. Proc. Natl. Acad. Sci. USA.

